# Time-on-task and instructions help humans to keep up with AI: replication and extension of a comparison of creative performances

**DOI:** 10.1038/s41598-025-05745-z

**Published:** 2025-06-20

**Authors:** Astrid Carolus, Martin J. Koch, Shuyan Feng

**Affiliations:** Institut Human-Computer-Media, Julius-Maximilians-University Würzburg, Oswald-Külpe Weg 82, 97074 Würzburg, Germany

**Keywords:** Creativity, Divergent thinking, Alternate uses task, Convergent thinking, Remote associates task, Artificial intelligence, Psychology, Human behaviour

## Abstract

A growing number of studies have compared human and AI creative performance. These studies differ in AI chatbots, human populations, creativity tasks, and creativity indicators (e.g., originality, usefulness, elaboration). They mostly neglect psychological research on determinants of creative performance such as instructions or processing time. The present study contributes to the theoretical foundation and replicates a study comparing humans’ and AI’s creative output in the Alternate Uses Task. Building on established knowledge of creativity determinants, we modified the Alternate Uses Task’s instructions (call for quality AND quantity), provided more time for the human participants, and added a second task (Remote Associates Task). The Alternate Uses Task output was scored in two ways: the mean and maximum scores of each Alternate Uses Task item, both in terms of semantic distances and in terms of human rating scores. The result shows that AI’s mean scores were significantly higher in the original and modified Alternate Uses Task condition, maximum scores in the original Alternate Uses Task condition, and in the Remote Associates Task. No significant differences between humans and AI were found for the maximum scores in the modified Alternate Uses Task. We mainly replicated the original studies’ findings. Our study provides initial clues that the evaluation of creative performances depends on creativity indicators and approaches (instructions and time).

**Supplementary Information:**

The online version contains supplementary material available at 10.1038/s41598-025-05745-z.

## Introduction

The fear and the debate that automation and technological progress challenge our human uniqueness and could, for example, make us obsolete in the workplace is not new^1,2^. However, with the rise of Artificial Intelligence (AI), this discussion has reached a new level^3–5^. Recent technological progress, especially in the area of Large Language Modelling (LLM), indicates that generative AI seems to approach creative capabilities that have been regarded as uniquely human. Even though earlier versions of AI already demonstrated creative capabilities (poems^6^ artworks^7^ and articles^8^), the release of OpenAI’s ChatGPT at the end of 2022 significantly fuelled the debate. Within a short period, generating surprisingly comprehensive creative output using ChatGPT became a mass phenomenon.

Koivisto and Grassini^9^ compared the creative abilities of human participants and AI in the form of divergent thinking abilities. Using the Alternate Uses Task, they found that the originality of the ideas generated by the AI was higher than that of humans. However, the study by Koivisto and Grassini has its limitations, such as the focus on originality in divergent thinking as the sole indicator of creativity and the task instructions focusing solely on the quality with a relatively short processing time of 30 s, which may have disadvantaged the human participants^10,11^and different sample sizes of humans (*n* = 256) and AI (*n* = 33). The present empirical study builds upon the study of Koivisto and Grassini^9^ by providing (1) a replication of their approach and (2) a modification of their approach, which addresses their limitations (i.e., longer processing times and modified instructions) with the idea to better unlock humans’ creative potential in the divergent thinking task. In a second study, we (3) added an expanded approach incorporating a convergent thinking task as an additional indicator of creative thinking abilities.

## Theoretical background

The following section establishes the conceptual foundations of ‘creativity’ and the approaches to study and measure it—for both target groups, humans and generative AI.

### Concepts of human creativity: divergent and convergent thinking

Creativity is a broad, multidimensional, and domain-specific construct^12,13^ lacking a unified and standardized definition^14,15^. Our study uses a widely accepted definition of creativity, focusing on two aspects: the ability to generate novel and useful ideas^14,16^. Guilford^17^ defined a total of 15 characteristics of a creative person, four of which are empirically well supported^18^: flexibility (i.e., the ability to break out of habit and transform the traditional into something new and different), fluency (i.e., the ability to generate many ideas and solutions), elaboration (i.e., the ability to work out details), and originality (i.e., the ability to generate novel and unusual ideas).

These four characteristics are reflected in divergent thinking. Tracing back to Guilford’s concept of divergent production^19–21^ as the ability to generate a wide variety of ideas that may thus lead to original and rare ideas, divergent thinking can be defined as the association of distantly related elements into new uncommon ones^16,22^. Thus, divergent thinking has been considered a key indicator of creative potential^13,19–21^. Convergent thinking is another concept of creative cognition, defined as the ability to produce the single best idea as a solution to a given problem^20,21^. Research emphasizes the effect of the interplay of both divergent thinking and convergent thinking on the creative outcome^23^.

### Measures of human creativity

As there is no uniform definition of creativity, measures of creativity vary^24^. Further, given the multifaceted construct of creativity, measures refer to specific domains of creativity rather than the construct as a whole, and measure results are not strongly correlated^25^. They cannot be easily interchanged^13^.

Snyder et al.^26^ distinguish four classes: divergent thinking and convergent thinking measures, self-report measures (e.g., self-assessment of creativity), product rating measures (i.e., expert assessment of the creative products, such as writing or drawing), and other measures. Barbot et al.^13^ suggest that divergent thinking tasks, product-based assessment, and self-report methodology are the three most used methods of measuring creativity. At the same time, many studies use novel methods to study creativity, such as research linking creativity and neuroscience to study the brain activity of convergent thinking and divergent thinking tasks^27,28^. Based on the review by Snyder et al., the prevalence of measures is changing. They identified a significant decrease in the use of divergent thinking and convergent thinking measures between 1984 and 2013 and an increase in the use of self-report measures and creative product measures. Despite that, divergent thinking and convergent thinking tasks accounted for over 42% of all the creativity measures in studies investigating creativity in undergraduate students. In their review, Benedek et al. showed that divergent thinking tasks alone accounted for over 51% of creativity measures in neuroscience research. In other words, divergent thinking and convergent thinking tasks remain the most used methods for measuring creative potential^18^.

Focusing on divergent thinking and convergent thinking measures, divergent thinking is commonly assessed with the *Alternate Uses Task*^19^ which consists of open-ended questions that ask people to generate as many creative ideas as possible for everyday objects such as rope or bricks. Regarding divergent thinking measured with the Alternate Uses Task, creative people also tend to generate more remote and less probable associated ideas^9,16,22^. The Alternate Uses Task has traditionally been scored regarding flexibility, originality, and fluency^29^. Referring to CT, Mednick introduced the *Remote Associates Test*^16^ as a measure of an individual’s ability to link seemingly unrelated items by finding a mediating relationship, such as a synonym or semantic association. In the Remote Associates Task, three cue words (e.g., *dream/break/light*) are presented, and—different from the Alternate Uses Task—participants are asked to give one correct response associated with the cue words (i.e., *day*).

### Measures of AI creativity

Compared to measures of human creativity, measures of AI creativity are not as diverse. Studies investigating the perceived creativity of products created or purportedly created by AI use product-based measures^30,31^. Thus, ratings of AI creative products are compared with ratings of human artistic work such as images, music, or short stories. Studies focusing on creative cognitions and comparing human versus AI creativity use divergent thinking measures such as the Alternate Uses Task^9^given its applicability to generative AI such as ChatGPT. Convergent thinking tasks have been very rarely used in the context of AI creativity^32^.

### Generative AI and creativity

Generative AI such as ChatGPT, as the current most popular application, can generate outputs, i.e., texts, images, or music. These outputs can be regarded as creative products, even awarded artwork^33^ or can be used for various creative purposes, from content creation to content enhancement and post-production workflows^34^. However, generative AI’s potential as a creator is still limited. First, AI technology is still evolving. Second, arts presumably created by AI were usually valued less than those of humans^30^ and very low purchase intention was shown for AI-created content^35^. Third, AI or LLMs are trained on existing data and expected to conform to pre-defined rules, which runs counter to the creative process of breaking out of routine behavior^34^.

The first empirical studies exploring the creative potential of (generative) AI directly compared its creative performance with that of humans. Since divergent thinking refers to the association of distantly related elements and transformation into new uncommon ones^16,22^ the concept indicates excellent potential for generative AI to outperform humans in divergent thinking tasks due to its vast memory capacities and fast access to large databases^9^.

The review of the studies so far reveals heterogeneous results. Some research found that although ChatGPT3 generated more useful ideas in the Alternate Uses Task, humans outperformed ChatGPT3 in all other creative indicators, such as originality, surprise of responses, and flexibility^36^. An expanded study used six AI chatbots (alpa.ai, Copy.ai, ChatGPT3 and 4, Studio.ai, and YouChat). It showed that the originality of the generated ideas was comparable between humans and AI chatbots^37^. Cropley compared the creativity of ChatGPT3.5 and ChatGPT4 with human norms on a verbal divergent thinking task, the Divergent Association Task (DAT, i.e., Generation of 10 words that are supposed to be semantically as distant as possible)^38^. The results showed that ChatGPT outperformed humans regarding mean DAT scores, with ChatGPT4 performing particularly better. This finding is consistent with the review of the average DAT scores of various AI tools, including GeminiPro, Bard, ChatGPT3, and 4, which showed that not all AIs could beat humans, with only ChatGPT4 outperforming humans and GeminiPro comparable to humans^39^. Hubert et al., who implemented three measures of divergent thinking (DAT, Alternate Uses Task, Consequences Task: i.e., generating as many consequences as possible for given hypothetical scenarios), arrived at similar results: ChatGPT4 performed significantly better than humans in terms of originality and elaboration on all three divergent thinking tasks^40^.

However, caution is advised when interpreting the results as evidence of AI creativity. First, despite the significant differences, the effect sizes can be relatively small^38^ at η_p_ = 0.01. Second, AI performance is less reliable than human performance because of repetitive responses and temporary unavailability due to too many inquiries^38,40^.

To summarize, studies in the context of AI creativity so far focus on divergent thinking. However, their empirical results are mixed due to differences in experimental approaches, operationalizations, creativity indicators, and evolving AI application versions. Furthermore, studies so far have revealed methodological limitations. First, samples are open to criticism due to limited sample sizes. For example, Stevenson et al. included only 42 native Dutch-speaking university students whose responses had to be translated from Dutch into English for a reliable scoring procedure^36^. Participants were primarily from the USA^37,40^ or, although from a larger sample, with a limited age range of 35–54 years^38^. Second, no tailored instructions were given to different AIs in different sessions^37,38^. To generate appropriate output, the task instructions need to be adjusted to each AI since tailored prompting (i.e., input instructions) is important to elicit desired outcomes from AI^41^. More generally, instructions affect task performance. Studies revealed that explicit instructions emphasizing creativity or quality and quantity improve divergent thinking performance^10,11^. Further, the confounding effect of fluency (i.e., the ability to generate many ideas and solutions) is a significant challenge for both the reliability and validity of divergent thinking tasks and the comparison of humans and AI^24,29,42^. Fluency contaminates the interrelationship between other divergent thinking indices, resulting in an artefactual correlation^42^. Regarding the sheer number of generated ideas and solutions, AI is massively advantaged due to its immense computational power compared to the limited cognitive resources of humans^34^. For example, Haase and Hanel did not control for fluency^37^. Moreover, the creativity evaluation of the output is mainly based on a subjective scoring method^40,43^ although recent research recommends automatic semantic distance scores for assessing originality due to their efficiency and high objectivity^22^. Finally, existing studies focused on divergent thinking and overlooked convergent thinking, typically using the Alternate Uses Task as their sole creativity measure^9,36–38,40^.

### The present study

Koivisto and Grassini overcome many of these limitations in their study^9^. With their focus on divergent thinking, they compared the Alternate Uses Task performances of a substantial sample of 256 humans and three AI chatbots. The instructions were adjusted as customized prompts to suit the AI chatbots and to adjust the number of responses to typical human response behavior. The prompts to the AI were adjusted to control for the chatbots’ preset number of responses (e.g., before the adjustment, ChatGPT3 always generated ten, ChatGPT4 seven to eight ideas) based on the median number of responses from the human participants. This specified instruction contributes to the challenge of fluency confound, which Koivisto and Grassini further addressed by controlling for fluency in their statistical analyses. To control for the difference in the average word counts of answers (chatbots tend to generate long responses, while humans generate one to three words responses), the maximum length of the chatbots’ answers was restricted. Alternate Uses Task results were scored in two ways: with a human “subjective rating” and objective scoring of semantic distances (using the open-source platform SemDis; semdis.wlu.psu.edu^44^). Further, they distinguished two scores of the Alternate Uses Task: mean scores (i.e., average scores of generated ideas for each object per person) and maximum scores (i.e., highest scores of each object per person). On average, chatbots outperformed humans in objective scoring of semantic distances and subjective human ratings. However, the top human ideas still equaled or surpassed those of the chatbots.

However, the study by Koivisto and Grassini still leaves room for improvement. (1) The Alternate Uses Task is their only indicator of creative thinking performance, which means that the study reflects divergent thinking but omits convergent thinking. (2) In their Alternate Uses Task instruction, participants were “asked to come up with original and creative uses for an object […], but creative quality is more important than quantity”. Thus, only creativity, not quantity, is emphasized. Additionally, participants were given a response time of 30 s, below the usually given two to three minutes, to generate creative ideas in the Alternate Uses Task^36,40,44^. Considering that AI chatbots generate rapidly fast outputs, the human inferiority in the approach of Koivisto and Grassini could result from slower human information processing and typing in of answers. Supporting this argument, Said-Metwaly et al. showed in their meta-analysis that long time limits significantly increased the originality scores in DT^11^. (3) Compared to the sample size of human participants (*N* = 256), the sample size of AI was relatively small (*N* = 33; three AI chatbots, 11 test sessions per chatbot). (4) Finally, there is another, more general challenge in this field of research. As AI technology is constantly evolving, the study’s results of the performance of AI chatbots can only be snapshots, applicable only to the specific chatbots examined.

To further explore the creative potential of AI, our study builds upon the approach of Koivisto and Grassini. To ensure the comparability of the results, a replication of their approach was conducted, supplemented with a modified version to address the original study’s limitations. (Alternate Uses Task instructions, response time, sample size of AI chatbots). Furthermore, the Remote Associates Task was added as an additional measure of creative thinking.

### Hypotheses and research question

The replication of Koivisto and Grassini’s approach should result in replication of their results. Thus, hypothesis 1 assumes the superiority of the chatbots in the Alternate Uses Task task:

#### H1

In the replication, AI chatbots outperform humans regarding creativity (originality) in the divergent thinking task Alternate Uses Task.

Addressing the limitations of Koivisto and Grassini results in the modified approach of our study in terms of Alternate Uses Task instructions, human participants’ response time, and the sample size of AI. Resulting in an approach that less strongly disadvantages human participants, hypothesis 2 assumes that the superiority of chatbots diminishes:

#### H2

In the modified approach, AI chatbots do not outperform humans regarding creativity (originality) in the divergent thinking task Alternate Uses Task.

Further, the modified approach widens the perspective on creative cognitions. It complements the Alternate Uses Task as the divergent thinking task with the Remote Associates Task, which assesses convergent thinking. Referring to the AI’s information processing velocity and memory capacity, superiority of AI chatbots for this second indicator of creative thinking is imaginable. However, because no empirical research exists to ground a hypothesis on, we instead ask a research question:

RQ1: Will AI chatbots outperform humans regarding creativity in the convergent thinking task Remote Associates Task?

## Empirical study 1

### Method: replication of Koivisto and Grassini (2023) and modified approach

The present study incorporates two parts: a replication of Koivisto and Grassini^9^ and a modified approach regarding modified Alternate Uses Task instructions. The two different Alternate Uses Task instructions were used for experimental manipulation.

Ethical approval of this study was not requested following the guidelines of the German Research Foundation (DFG, https://www.dfg.de/de/foerderung/antrag-foerderprozess/faq/geistes-sozialwissenschaften). For studies of all social sciences, including research with human participants, ethical approval is only required when using identifiable data, when including patients or other vulnerable groups, when using material that elicits strong emotion (e.g., stress or traumatic experiences) or physical danger or pain, when the participants are not informed about the aim of the study or are deliberately misled as experimental manipulation, or are exposed in any other way to special social, legal, financial, or professional risks. In addition, for psychological studies, ethical approval is required when using electric or magnetic stimulation or psychopharmacological examinations. Risks and harm to the participants are not to be expected in the surveys and tests on non-sensitive topics we conducted. The privacy rights of human subjects have been observed and informed consent was obtained from all participants. Thus, all methods were carried out in accordance with relevant guidelines and regulations.

### Sample: human participants and AI chatbots

In total, 100 individuals (48% female, 51% male, 1% not known) aged 19–74 years (*M* = 37.04, *SD* = 13.10) with English as a first language from different countries including the UK (51.00%), South Africa (10.00%), the US (10.00%) and Canada (7.00%) participated in the study. One half received the original Alternate Uses Task approach as used by Koivisto and Grassini (*n* = 50; 48% female, 50% male, 2% not known; aged 21–70 years, *M* = 36.70, *SD* = 13.48), the other half received our modified approach (i.e., modified instructions and longer processing time) (*n* = 50; 48% female, 52% male; aged 19–74 years, *M* = 37.38, *SD* = 12.83).

The AI sample (*N* = 93) consisted of four different AI chatbots: Open AI’s ChatGPT (version 3.5 and 4) and CopyAI as in the original study complemented by Google’s Bard (now Gemini). In the original Alternate Uses Task condition, each chatbot “participated” 11 times (*n* = 33). However, as Bard has not generated any output corresponding to the prompt, Bard was excluded. In the modified version, all four chatbots “participated” 15 times (*n* = 60).

### Measures of divergent thinking

Original instruction by Koivisto and Grassini^9^ for human participants: Alternate Uses Task was used to measure creative thinking. Following Koivisto and Grassini, for each object (rope, box, pencil, and candle), participants were asked to “come up with creative ideas” defined as “clever, unusual, interesting, uncommon, humorous, innovative, or different” and they were reminded that “creative quality is more important than quantity”.

Original instruction for AI chatbots: Due to the characteristics of AI chatbots, the instructions to the AI were the same as to the human participants, except that (1) the number of ideas and (2) the number of words in the output was limited in each session for each object. Accordingly, the AI was instructed to “type in one [or two/three/four/five/six] ideas” and “use only 1–3 words in each response”. One, five, and six answers were used 2 times each. Two and four answers were asked two times each, and three answers were requested four times.

Modified instruction for human participants: Compared to the original Alternate Uses Task as used by Koivisto and Grassini, three changes were made: (1) Human participants received 2 min instead of 30 s to generate (2) as many ideas as possible. (3) The instructions suggested that creativity and the number of answers were equally relevant to the task, “both creative quality and quantity matter”. We increased the time following research that shows that longer times improve the performance^11^. We have changed the instructions following findings that suggest that explicit instructions emphasizing quality and quantity improve performance when compared to instructions that only emphasize quality^10^. Modified instruction to AI chatbots was the same as the modified instruction to humans. The original and modified instructions for humans and AI can be seen in the appendix (Appendix A).

### Procedure

A gender-balanced sample of individuals with English as a first language was recruited online via Prolific.co^45^ on January 16th, 2024, and directed to the study on soscisurvey^46^ hosted on a university server. After reading the introduction and informed consent was obtained from all participants. Participants completed the Alternate Uses Task. The original approach of Koivisto and Grassini^9^ was followed for the replication. The modified approach used modified instructions (see Appendix A for the wording) and a prolonged response time (2 min per task). Finally, sociodemographic information was queried. The average completion time in the original condition (*M* = 12.46, *SD* = 3.12 min) was shorter than in the modified condition (*M* = 17.95, *SD* = 2.49 min), mainly due to the longer processing time for the Alternate Uses Task task. Participants received 2.70£ (original approach) or 4.50£ (modified approach).

Alternate Uses Task tasks were presented to the AI chatbots from January 12th to 21st, 2024. Between sessions, longer breaks were necessary due to technical challenges such as limited usage times and access to the AI chatbots. Chatbots’ Alternate Uses Task outputs were manually collected. No further information was collected.

### Analysis

#### Data cleaning and semantic distance

Human participants’ data were cleaned manually before being analyzed. Expressions such as “DIY”, “to make as”, “to use as”, and “making” and stop words such as “or”, “and”, and “to” were deleted (e.g., “use as a DIY dog lead” was changed to “dog lead”) and hyphens replaced dashes. The AI output needed further cleaning as the chatbots usually answered in complete sentences, sometimes in tables or pictures or with accompanying explanations. Cleaning was especially important for the modified instructions where the scope and form of answers varied more strongly. The core statements were broken down into a few words to allow comparison with human answers. No word of the core statement was omitted, no wording was changed, and no new words were generated not to impact the originality scoring (except for picture descriptions). Sometimes, in addition to the answer itself, the AI gave detailed explanations – which were not considered for originality scoring. Sometimes, the AI produced a different number of answers than the prompt required (relevant for the original approach only), or the output generation was aborted. In these cases, prompts were repeated. Following Koivisto and Grassini, the originality of an answer was operationalized as the semantic distance between the given object and the generated answer. It was calculated with SemDis^44^. To ensure comparison with the original study, the SemDIS settings were adopted: the multiplicative compositional model was chosen to account for responses with multiple words, the setting to remove filler words and clean the data (e.g., remove *the*,* an*,* a*,* to*,* and* punctuation marks) were copied, and five different models were used, and their output is combined into a final originality score for each answer. Average and maximum scores for each task separately were calculated for each participant (i.e., individual human participant and AI session).

#### Subjective ratings

Three human raters scored subjective originality. The raters received training in which the Alternate Uses Task instructions were explained (instructions for human participants, prompts of the chatbots). Based on Koivisto and Grassini, they received the same coding examples (“cutting” in response to the object s*cissors* as being unoriginal and uncreative). In a trial session, test coding was conducted, and questions and uncertainties were addressed and discussed. The raters were unfamiliar with the aim of the study and did not know whether the Alternate Uses Task responses came from humans or chatbots to avoid bias. They were asked to rate creativity on a 5-point Likert scale (1 = *not creative at all*, 5 = *very creative*) using the original instructions as an evaluation standard. Further, they were explicitly instructed that novelty is more important than usefulness and that confusing or illogical answers are to be rated with a 1. One example was given.

The order of responses and objects was different for each rater. For example, Rater A started with the object Rope, and the responses were presented from A to Z. Rater B started with the object Candle, and the responses started with the letter Z, and so on. Each response was rated by two raters. We assessed the interrater reliability at the response level (i.e. for each response, the ratings were set in relation to each other) using Krippendorff’s α. The overall interrater reliability was low for the whole group (α = 0.41), and higher for humans (α = 0.48) compared to AI (α = 0.30). The interrater reliability for the single tasks was not sufficient as well (ICC_pencil_ = 0.60, ICC_box_ = 0.35, ICC_candle_ = 0.52, ICC_rope_ = 0.44). They differ between the AI (ICC_pencil_ = 0.64, ICC_box_ = − 0.05, ICC_candle_ = 0.38, ICC_rope_ = 0.14) and the human participants (ICC_pencil_ = 0.56, ICC_box_ = 0.50, ICC_candle_ = 0.63, ICC_rope_ = 0.15). Because of the low internal consistencies, we did a second round of rating with other raters (*n* = 2). They did a binary rating of whether an answer was illogical/nonsensical. Cohen’s Kappa was 0.39, which is considered to be fair^47^. Then, each rater had a look at the other’s ratings and could change their opinion. The remaining discrepancies were discussed and resolved. 247 of 6965 answers’ ratings were set to zero to represent that they are nonsensical. Of these, 156 were given by humans and 91 by AI. For the other answers, the average ratings across the three raters from the first round were kept. The subjective ratings represent the subjective nature of the perception and evaluation of creative performance.

The mean and maximum scores for each Alternate Uses Task (i.e., object) were calculated for each participant. The descriptive statistics for each group and task are in the Appendix (Table B1 and Table B2).

#### Fluency

Fluency was calculated as the number of responses a participant gave across all four tasks of the Alternate Uses Task. Included were all responses that remained in the data after the cleaning of the data. This includes the items that were removed because they were considered illogical/nonsensical.

#### A-priori power analysis

We conducted an a-priori power analysis using the effect sizes of Koivisto and Grassini as our basis. We computed the effect size d for all four measures (mean and maximum semantic distance and subjective rating) using the means and standard deviations they reported. We approximated the necessary sample size for all four measures’ average and smallest effects using G*Power^48^ with a linear approach (t-test) instead of a mixed model. We chose this approach because we were unaware of unique approaches to calculating power for linear mixed models^49^. To achieve an α = 0.05 and a power (1−β) = 0.80, a sample size of 28 humans and 28 AI would be necessary to replicate their average effect. A sample size of 48 humans and 48 AI would be necessary to replicate the smallest effect. By including a higher number of individuals for each group (*n* = 100 humans, *n* = 93 AI), we ensured that our power was high enough also to find effects in the subgroups (i.e., original and modified approach).

#### Data analysis

We ran linear mixed model analyses using JASP Version 0.18.3.0^50^. We included the mean and maximum semantic distance and mean and maximum subjective rating for all answers given to an object as the dependent variable. Because we wanted to test the influence of the approach (original vs. modified) and the group (human vs. AI) on the dependent variable when controlling for fluency, we added fixed effects of the approach (original = 0 vs. modified = 1), group (human = 0 vs. AI = 1), and fluency (covariate). Positive β-values would indicate higher values for the modified approach or the AI, respectively. We also included the interaction of the approach and group to see how the two factors influenced each other’s effect on the dependent variable. In case of a significant interaction, we calculated conditional effects (i.e., conditional effects of the approach for both groups and conditional effects of the group for both approaches). The conditional effects of the group for the original approach test hypothesis H1, while the conditional effect of the group for the modified approach tests hypothesis H2. The participant ID was included as a random effect grouping variable. This results in the following model(s): dependent variable ~ approach + group + approach x group + fluency + [1|participant], with the dependent variable being the mean and maximum semantic distance and mean and maximum subjective rating in one analysis each. The data, thus, consisted of 765 data points clustered among 193 participants. As different models were not compared, no model fit indices were reported (e.g., deviance, AIC, BIC). Significance was calculated using Satterthwaite’s method to estimate degrees of freedom and generate *p*-values for mixed models with square sums type III.

We decided not to replicate the ANOVA by Koivisto and Grassini for two reasons. First, referring to classical test-theoretical approaches, only the combination of the Alternate Uses Task tasks – not viewing them separately – constitutes a valid measure of divergent thinking. Second, we aim to compare humans and AI participants as groups, not individually. However, the ANOVA is included in the supplementary material (supplement I) for completeness. In addition, there is a comparison of the variance (i.e., absolute mean differences) using linear mixed-effects regressions with approach and group as fixed effects (supplement II).

### Results

**Fig. 1:**
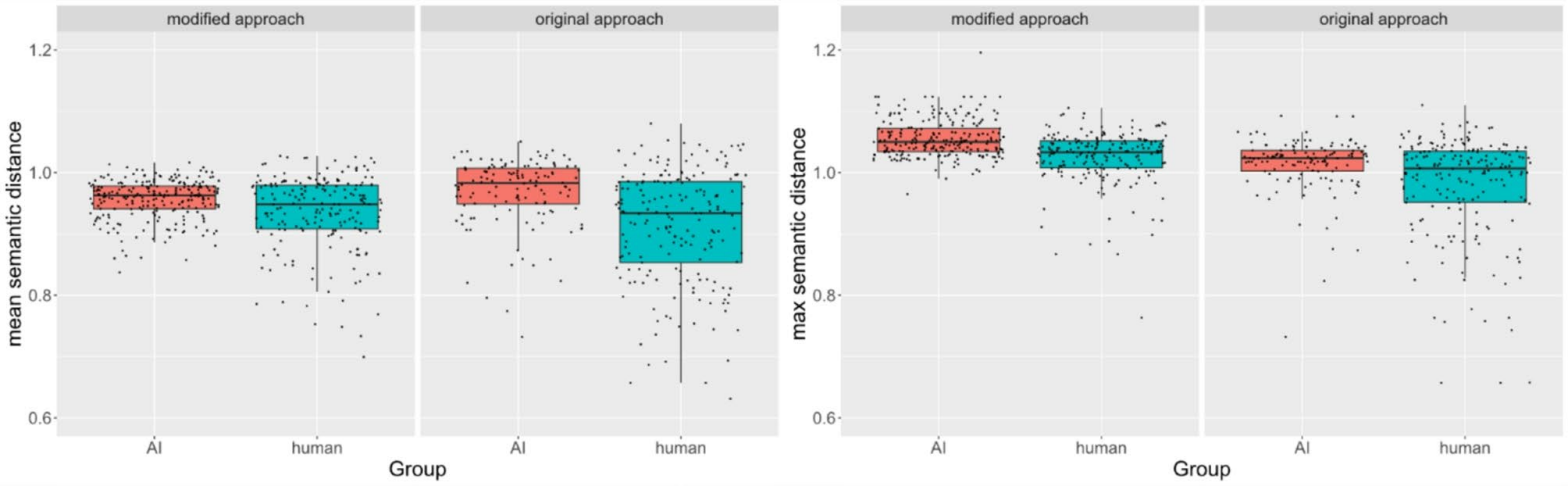
Boxplots for the semantic distance separated by measure, group, and approach.

First, we included the mean semantic distance as the dependent variable (Fig. 1). The first model revealed a significant main effect of the group (AI vs. human) on the mean semantic distance. On average, AI chatbots gave answers with a significantly higher mean semantic distance than humans. We did not find effects of fluency or approach or a significant interaction of approach and group (Table 1).

**Fig. 2:**
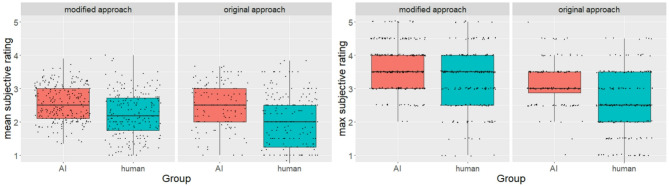
Boxplots for the subjective rating by measure, group, and approach.

The analysis was repeated with identical settings for the maximum semantic distance as a dependent variable (see Fig. 2). While the main effect of the approach was not significant, a significant effect of fluency on the maximum semantic difference and a significant main effect of the group (AI vs. human), as well as an interaction of the approach and the group, was found (Table 1). Because of the significant interaction, we tested for conditional effects of the experimental variables. We found a simple effect of the approach on the maximum semantic difference only for humans but not for the AI (Table C1). We also found a simple effect of the group on the maximum semantic difference for the original approach but not for the modified approach (Table C2). These results indicate that humans and AI differed when they received the original approach – with AI chatbots showing significantly higher values than human participants. Compared to humans who received the original approach, humans with the modified approach showed significantly higher maximum semantic distance scores on average. This difference was not significant for the AI chatbots leading to no significant difference between humans and AI under the modified approach.

Second, we repeated the mixed effects analysis and included the mean subjective ratings (Fig. 2) as a dependent variable. The group (AI vs. human) significantly affected the mean subjective rating. Identical to the mean semantic distance, we neither found effects of the fluency or the approach nor a significant interaction of approach and group (Table 2). On average, AI gave answers with a significantly higher mean subjective rating than humans.

We also computed the mixed effects analysis using the maximum subjective ratings (Fig. 2) as a dependent variable. Results revealed a significant effect of fluency on the maximum subjective rating and a significant interaction between the approach and the group. The main effect of the group was also significant (Table 2). Because of the significant interaction, we tested for conditional effects of the experimental variables. We found a simple effect of the approach on the maximum subjective rating only for humans but not for the AI (Table C3). We also found a simple effect of the group on the maximum semantic difference for the original approach but not for the modified approach (Table C4). These findings parallel the effects for the maximum semantic distance: (1) Only for the original approach did humans and AI differ - with higher values for AI. (2) For the modified approach, humans reached higher values than the original approach – the AIs’ maximum subjective ratings were unaffected by the approaches. Human and AI, thus, did not differ under the modified approach.

**Table 1:** Results of the mixed linear model on the mean and maximum semantic distance.

Fixed effects	Mean semantic distance					Maximum semantic distance		
	β	*t*	*df*	*p*	β	*t*	*df*	*p*
Intercept	0.96	93.81	188.44	< 0.001	0.99	122.12	188.34	< 0.001
Fluency	0.00	− 1.46	187.78	0.145	0.00	3.21	187.55	0.002
Approach	0.01	1.65	187.30	0.100	0.01	1.96	187.01	0.051
Group	0.02	5.75	186.82	< 0.001	0.10	3.26	186.43	< 0.001
Approach × group	− 0.01	− 1.34	188.47	0.181	− 0.01	− 2.26	188.42	0.025

Random effects	*Var*	*SD*			*Var*	*SD*		
Participant	0.00	0.03			0.00	0.02		

**Table 2:** Results of the mixed linear model on the mean and maximum subjective ratings.

Fixed effects	Mean subjective rating				Maximum subjective rating			
	β	*t*	*df*	*p*	β	*t*	*df*	*p*
Intercept	2.34	23.96	185.91	< 0.001	2.68	22.477	187.49	< 0.001
Fluency	− 0.00	− 0.33	185.10	0.741	0.02	4.064	186.72	< 0.001
Approach	0.10	1.76	184.54	0.080	0.08	1.214	186.17	0.226
Group	0.23	6.04	183.80	< 0.001	0.16	3.587	185.47	< 0.001
Approach × group	− 0.04	− 1.17	185.74	0.243	− 0.16	− 3.593	187.34	< 0.001

Random effects	*Var*	*SD*			*Var*	*SD*		
Participant	0.05	0.23			0.09	0.31		

## Empirical study 2

### Method

The study procedure for study 2 was identical to study 1. However, instead of using the Alternate Uses Task as a task for divergent creative thinking, we used the Remote Associates Task for convergent creative thinking. The average participation time was 27.11 min (*SD* = 3.01), for which participants received 6.30 £. The AI data were collected on Friday 12th, and January 13th, 2024. Ethical approval of this study was not requested following the rationale outlined for study 1.

### Measure: the remote associates tasks

We used a 30-item version of the Remote Associates Task^51^ to measure convergent creative thinking. We used the same 30 items that were used by Lee et al.^52^who selected items from an RAT item bank^52^ to present a wide range of easy to difficult items. In their study, the number of participants solving the items in 15 s ranged from 11 to 76%. Participants were given practice item sets in four practice trials with their respective solutions. The Remote Associates Task presented three cue words (e.g., flake/mobile/cone) and a blank response field. Participants had 15 s to type the solution word (e.g., snow) before the next item set appeared. Unlike the Alternate Uses Task, we are not aware of a discussion about the time limit for the Remote Associates Task. Thus, we decided to keep the time limit from the Remote Associates Task’s study^52^. The 30 items were presented in ascending order of difficulty. If an item was answered correctly, a one was assigned. If not, a zero was assigned. A total score of correct answers was calculated for each participant.

### Sample

A total of *n* = 50 people (50% female, 50% male) aged 20–71 years (M = 38.90, SD = 14.42), with English as their first language, from various countries including the UK (60.00%), Canada and South Africa (12.00% each), the USA (8.00%) participated. Four AIs (Bard, CopyAI, ChatGPT 3.5, ChatGPT 4) “participated” in the Remote Associates Task, each in ten sessions, for *n* = 40 chatbots.

### Analysis

First, to mirror the analysis from study 1, we conducted an independent sample t-test to analyze differences between the average Remote Associates Task scores of the human and the chatbot group. These differences were further analyzed in an ANOVA with the between-subject factor group (CopyAI, ChatGPT3.5, ChatGPT4, and humans). Mean values and standard deviations can be seen in Table 3.

**Table 3:** Mean scores and standard deviations for each group for the results of the remote associates task.

	M	SD
BardAI	3.90	3.81
CopyAI	20.40	4.65
ChatGPT3.5	23.60	0.97
ChatGPT4	24.20	0.42
AI total	18.03	8.89
Human	13.24	6.22

### Results

**Fig. 3:**
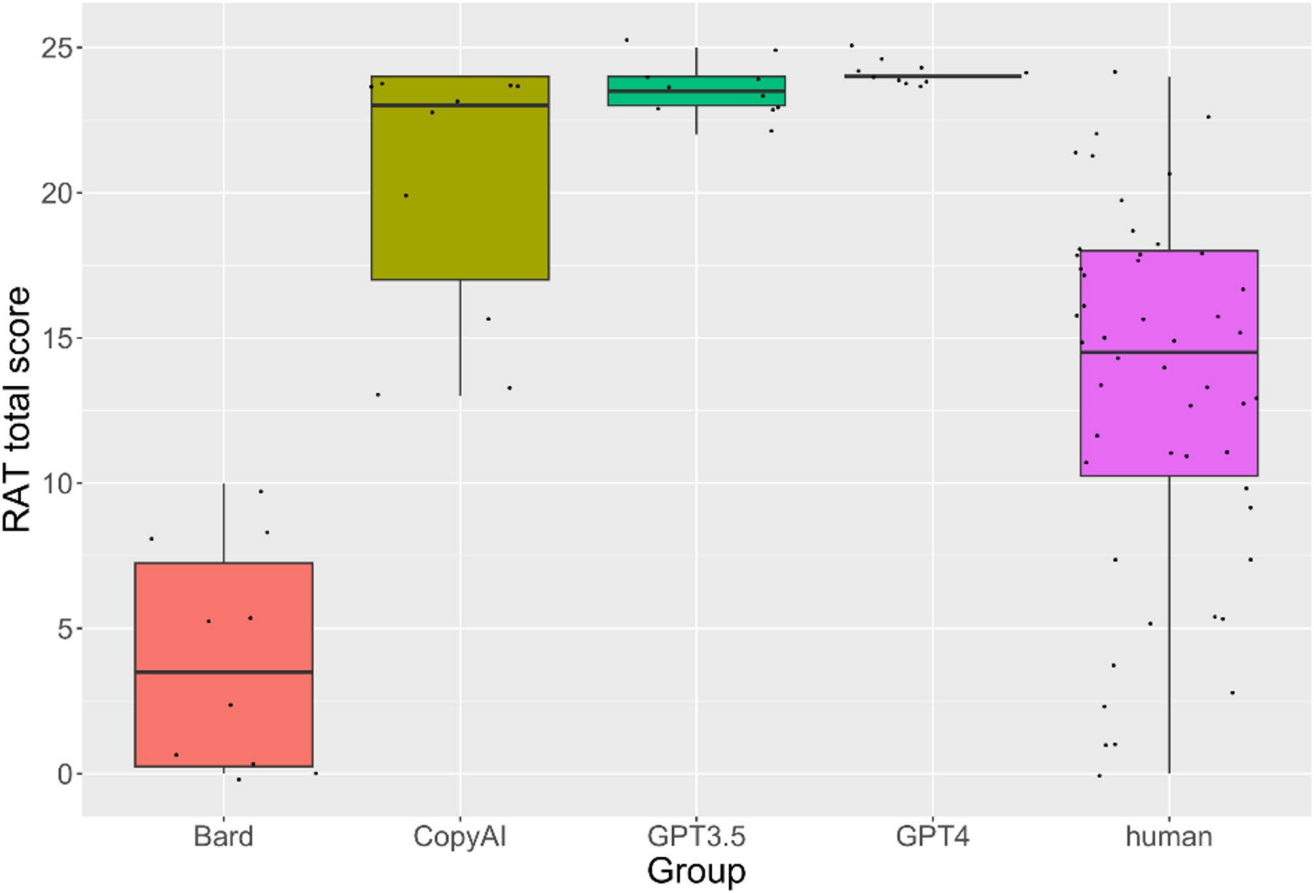
Boxplots for the remote associates task total score for the AI types and human groups.

We found a significant mean difference (*t*(88) = 3.00, *p* = .004) revealing that AI chatbots scored significantly higher (*M* = 18.03, *SD* = 8.89) than humans (*M* = 13.24, *SD* = 6.22; Fig. 3).

Because Levene’s test indicated a difference of variances (*F*(4,85) = 8.36, *p* < .001), and because a Q-Q plot indicated only minimal deviations from a normal distribution, we used a Welch correction for the ANOVA. We found a significant effect of the group on the total Remote Associates Task score (*F*(4, 24.87) = 99.57, *p* < .001, η² = 0.59), indicating differences between at least two of the groups. Holm-corrected post hoc tests showed several statistically significant results (Table 4). The average scores of Bard are significantly lower than the scores of all other AIs and the human group. Further, the human group showed significantly lower average values than CopyAI, ChatGPT3.5, and ChatGPT4.

**Table 4:** Post-hoc differences for the remote associates task.

		M_diff_	SE	t	*p*_holm_
Bard	CopyAI	− 16.50	2.29	− 7.21	< 0.001
	ChatGPT3.5	− 19.70	2.29	− 8.60	< 0.001
	ChatGPT4	− 20.30	2.29	− 8.87	< 0.001
	Human	− 9.340	1.77	− 5.27	< 0.001
CopyAI	ChatGPT3.5	− 3.20	2.29	− 1.40	0.332
	ChatGPT4	− 3.80	2.29	− 1.66	0.302
	Human	7.16	1.77	4.04	< 0.001
ChatGPT3.5	ChatGPT4	-0.60	2.29	-0.26	0.794
	Human	10.36	1.774	5.84	< 0.001
ChatGPT4	Human	10.96	1.774	6.18	< 0.001

To explore whether the difficulty of the items affected the scores of human and AI participants, we grouped the items based on the a-priori difficulty in three sets of 10 items and calculated separate scores for each of the item difficulties (low, medium, and high difficulty). We used a two-way ANOVA with the between-subject factor group (AI and humans) and the within-subject factor item difficulty (low, medium, and high difficulty). We found a significant effect for the difficulty (*F*(2, 176) = 32.20, *p* < .001, η² = 0.05) and the group (*F*(1, 88) = 6.07, *p* = .016, η² = 0.05). The interaction was only marginally significant (*F*(2, 176) = 2.61, *p* = .076). We nonetheless decided to test for conditional effects. We found that AI scored significantly higher than humans for the high difficulty (*t*(88) = 3.19, *p* = .002) and medium difficulty *t*(88) = 2.72, *p* = .008) but not for the low difficulty items (*t*(88) = 1.26, *p* = .210). Humans (*F*(2, 98) = 16.42, *p* < .001, η² = 0.25) scored significantly lower in the medium difficulty compared to the low difficulty and in the high difficulty compared to the medium difficulty (all *p* < .01). AI (*F*(2, 98) = 36.13, *p* < .001, η² = 0.48) scored identical in low and medium difficulties (*p* = .755) but scored significantly lower in the high difficulty items compared to the other two item groups (both *p* < .001).

## Discussion

The study compared AI and human creative capabilities in convergent and divergent thinking tasks. In study 1, we replicated and modified the approach of Koivisto and Grassini (2023), who compared the performances of human participants with the performance of AI chatbots in the Alternate Uses Task as a measure of divergent thinking. In study 2, we used a different task (Remote Associates Task) on creative capabilities to compare human and AI participants.

### Summary and interpretation

Study 1 replicated the approach of Koivisto and Grassini as closely as possible. Because we considered their Alternate Uses Task instructions and available time to be at a disadvantage to human participants, we modified their approach following well-established practices^10,11^. Further, we included Google’s Bard AI in the analysis. However, with the original prompts, Bard did not generate any responses.

Considering the mean semantic distance and subjective ratings, we found that, on average, AI scored higher than the human participants – both for the original and the modified approaches. Considering the maximum semantic distance and subjective ratings, the AI score was significantly higher than that of humans – but only for the original approach and not for the modified one. In the modified approach, humans achieved significantly higher average maximum semantic distance scores and maximum subjective ratings than in the original approach. This difference was not significant for the AI. The higher score for humans in the modified approach leads to a non-significant difference between humans and AI for maximum semantic distance and maximum subjective rating.

To conclude, human participants benefited from the modified approach: their maximum semantic distance and subjective ratings were not significantly inferior to the chatbots’ results. On average, AI can produce better results in the Alternate Uses Task. Modifying the parameter of the Alternate Uses Task to be more advantageous for the human participants increases their performances, at least for the maximum semantic distance and subjective ratings. In contrast, the AIs’ performance remained unaffected.

Complementing the analysis of divergent thinking, study 2 widens the approach by using the Remote Associates Task, which measures the ability to find the correct answer to a given task. Consequently, the Remote Associates Task and Alternate Uses Task pose qualitatively different demands to human thinking and AI computing as a prerequisite of creativity. Results revealed that, while Bard could barely solve the Remote Associates Task, all other AIs were superior to humans, indicating superiority in convergent thinking.

### The role of AI functioning

The way generative AI works can provide helpful insights that need to be considered when interpreting the results. It seems highly likely that LLMs, as part of their training data, had access to the previous responses of humans to the Alternate Uses Task and Remote Associates Task items. Better AI scores can, therefore, possibly be explained by “familiarity” with the tasks. AI could, therefore, possibly benefit from the fact that it was trained with data from a vast set of potential responses based on prior participants’ responses. It seems difficult to interpret creativity and originality in relation to AI, as remembered responses might play an important role in their performance.

Further, it seems reasonable to assume that generative AI performs well in the Remote Associates Task, as their functionality is explicitly designed to predict the next language building block (token), considering the previous information. The AI shows very consistent performance, and there already seem to be ceiling effects (apart from Bard). The theoretical implications of our findings might be limited as generative AI continues to improve.

### Comparison with prior findings

Our findings in the original approach of study 1 and of study 2 are mostly consistent with the findings of Koivisto and Grassini (2023). In line, other prior studies showed that AI is more creative than humans considering usefulness^36^divergent associations^38^or originality and elaboration^40^. In the modified approach of the current study, the results support the notion that there are no differences between human and AI performance regarding originality, as shown by Haase and Hanel^37^. However, the findings did not show that humans are better than AI (as shown for originality, surprise of responses, and flexibility^36^), except that humans outperformed Bard on the Remote Associates Task in study 2.

Contrary to Koivisto and Grassini (2023), the best humans did not outperform the AI. The highest values for humans and AI were very similar for semantic distance (1.11 vs. 1.20) and Remote Associates Task (24 vs. 25), and identical for the subjective rating (5 vs. 5).

As described in the theoretical background of this paper, several factors limit the validity and generalizability of the existing results, such as small effect sizes, limited sample sizes, different task instructions for (different) AI and humans, and an over-focus on Alternate Uses Task.

### Determinants of the differences between ai’s and humans’ performance

There are various reasons for the different findings in the previous research: our current study shows that the task and the chosen approach (i.e., instructions and available time) are important factors determining the superiority or non-superiority of AI or humans. By using modified instructions that focused on quantity and creative quality^10^ and offering a longer time for each item of the Alternate Uses Task^11^we could increase human performance without limiting the AI. Our findings strongly suggest that each prior comparison of AI and human creative performance needs to be viewed in the light of the chosen approach. Differential findings might be conditional on the selected approach.

We decided to use the time constraint for the Remote Associates Task described by Lee et al.^52^ (15 s to type the solution word). This constraint may have contributed to the group difference, particularly to the AI advantage. While LLMs can generate quick responses, the human group seems to have a greater need for time to answer. This idea is supported by our exploratory finding that humans and AI only differed in the Remote Associates Task when considering high- and medium-difficulty items but not for the low-difficulty items.

Moreover, the studies differ in the AI chatbots that were included. We expanded the selection of chatbots by Koivisto and Grassini (2023) and used different versions of ChatGPT, Copy AI, and Google’s Bard, which are based on different data sources^53^. Stevenson et al. (2022) only used ChatGPT 3, and Cropley (2023) only used ChatGPT 3.5 and 4, whereas, in the study by Haase and Hanel (2023), six different chatbots were used (alpa.ai, Copy.ai, ChatGPT3 and 4, Studio.ai, YouChat)^36–38^. The selected chatbot’s importance is highlighted by Bard AI’s results in our study. Google’s Bard was not able to generate answers for the original instructions of the Alternate Uses Task and solved the Remote Associates Task only poorly. This highlights the large differences between chatbots. Further, shortly after the study, Google announced Gemini as the successor to Bard, emphasizing the rapid changes in the area, which dramatically challenges researchers. In the same spirit, it needs to be discussed that the studies collected data at different points in time. AI models change over time due to updates and learning patterns that change the processes and affect the output to identical prompts. The creative performance of the same models thus can change. AI can get better or even worse at solving different tasks of creative “thinking”.

Similarly to the selection of chatbots and the time frame of the study, the studies differ from ours in their selection of tasks (e.g., Alternate Uses Task, Remote Associates Task, DAT) and operationalizations of creative output (i.e., originality, elaboration, or usefulness). These selections might also heavily impact the results of the chatbots.

Lastly, the human samples might also limit the representativeness of prior studies. They use national samples^9,36,37,40^ and only include participants of a limited age range^9,38^. We address these issues by including a rather international sample from different English-speaking countries (mainly but not only the UK, South Africa, Canada, and the USA) with a wide age range of participants, from young adults to seniors, limiting the age effect on creative cognition^54^.

Our sample was collected online without any specification, so our findings were obtained from a general population sample. In certain sub-populations, the findings might be vastly different: people with artistic professions (e.g., designers, draughtsmen, marketing) and hobby artists (e.g., musicians, authors, painters) could perform better at the tasks we choose and thus match or even surpass AI.

### Limitations

Performance on Alternate Uses and Remote Associates tasks may not reflect real-world creative performance. It is possible that these or similar tasks are part of the AI’s training data, while new tasks requiring professional-level creative thinking may be more difficult for AI.

Several methodological limitations of the present study need to be mentioned. Firstly, the power analysis does not entirely reflect the reality of our chosen method in the current paper. While the power analysis was conducted a-priori and while it was based on a linear model (i.e., t-test), we used a mixed-effects model which combines linear effect for the group, the approach, their interaction, and the covariate fluency with a random effect for the participant. We chose a sufficiently larger sample size to make up for this limitation, especially the additional condition we added in the form of the modified approach.

As Bard AI failed in the Alternate Uses Task using the original approach of Koivisto and Grassini (2023), we decided not to include the measures of the original approach for Bard AI in our analyses. An alternative approach to this issue would have been to enter all Bard AI scores as zero since the AI did not give any creative answers or to change the prompt to enable Bard AI to give responses. We, however, decided as we did as we felt it to be the “fairest” option for the AI and the human group. We recruited our participants using an online panel (Prolific). While these panels are often used for online surveys or questionnaire studies, their use seems to be less common in experimental studies. Presumably, our online experimental study required high effort from participants. Thus, human results in our tasks might be negatively impacted by limited effort. The human participants may have lacked motivation and effort. Human participants may have completed the tasks in a low-effort manner simply for the reward. While this is not a major limitation of the current results, human participants may perform better on tasks that are more engaging, further reducing the differences between human and AI participants.

Compared to Koivisto and Grassini, our procedure for the subjective ratings was shortened to increase efficiency. Two out of three raters rated each item of one task (compared to the six raters in the study by Koivisto and Grassini). This may have come at the expense of precision in terms of the reduced interrater reliability of our coding (low Krippendorff’s α and ICCs). Alternatively, low interrater reliability can also be explained by the rather imprecise instruction and the short training the raters were provided with. Our raters were trained using a short PowerPoint presentation. This contained information on the Alternate Uses Task (e.g., goal, instructions, and items), the number of responses and participants, the guidelines for rating, and an example item and response. Our training replicated the instructions from the original study as closely as possible. Following Koivisto and Grassini: “instruction stressed that they should stress novelty over usefulness and use the instruction given for participants as the reference point against which to evaluate the responses” (p. 3), building upon raters’ different perceptions and feelings. Our training may not have been extensive enough to ensure a consistent rating of each rater. It also seems possible that individual raters may have understood or interpreted instructions differently. In addition, the rater’s lack of experience may have led to overrating problems. They were student assistants without any prior experience in rating the Alternate Uses Task or other creativity assessment tasks. Further concerning our measures, as already discussed, we only used originality as an operationalization of creativity in the current study using the Alternate Uses Task, not considering other indicators of creativity such as flexibility, fluency, usefulness, or elaboration.

We used the time constraint for the Remote Associates Task described by Lee et al.^52^ (15 s to type the solution word). As our results indicate, it would have been necessary to extend the time frame.

### Future research

Future research should continue to focus on the methodological aspects of analyzing AI’s creative capacities. To gain robust insights into the potential of this technology, established quality criteria for scientific analysis must be addressed. For example, studies should systematically vary the operationalization of creative processes (e.g., divergent vs. convergent thinking), the methodological approach (e.g., Alternate Uses Task or Remote Associates Task), and task conditions (e.g., instructions, processing time), as well as the selected human sample (e.g., age, education, cultural background, or sub-samples of (professional) artists or creatives) and the AI chatbots (e.g., from different providers and in various versions). For the Remote Associates Task in particular, time should be examined more systematically. Especially newly developed chatbots and progress in existing AI chatbots might lead to vastly different results in the future. Only by systematically varying these factors will it be possible to establish a research area that provides meaningful insights into the differences in creative performance between humans and AI. Future research should also address the potential limitation of low motivation in human participants and a potential advantage of AI resulting from training with Alternate Uses and Remote Associate Tasks by using meaningful real-world tasks.

### Conclusion

The replication and modification of Koivisto and Grassini’s (2023) study provide insights into both human and AI creative performance, as well as the methodological challenges of measuring creativity. Our results highlight the importance of carefully considering how creativity is conceptualized and measured, demonstrating that differences between human and AI performance depend on several factors: creativity indicators (e.g., maximum vs. average scores per task), the approach (i.e., specific instructions and processing time), and the operationalization of creativity (e.g., Alternate Uses Task vs. Remote Associates Task), as well as the type of analysis conducted. For the Alternate Uses Task, we observed that humans and AI perform similarly on creative tasks when maximum scores are used with a modified approach (i.e., instructions emphasizing both quality and quantity of results and a 2-minute processing time). In contrast, we identified significant differences when using alternative scoring methods, approaches, and tasks.

In sum, our study contributes to an emerging field of empirical research by providing insights into the importance of carefully considering the theoretical conceptualizations of creativity and the methodological approach to measure creative performances. In this context, our study provides the first evidence that task instructions and the time to solve the task are key factors in differentiating human and AI creative performance. Ultimately, our research makes a significant contribution by showing that boundary conditions and selected measures are crucial when comparing creativity between humans and AI.

## Electronic supplementary material

Below is the link to the electronic supplementary material.

Supplementary Material 1

## Data Availability

Data will be made available upon reasonable request. Please contact the corresponding author.

## Appendix 1

### Modified instructions for the alternate uses task

Humans: For the next task, you’ll be asked to come up with original and creative uses for an object. The goal is to come up with creative ideas, which are ideas that strike people as clever, unusual, interesting, uncommon, humorous, innovative, or different. Your ideas don’t have to be practical or realistic; they can be silly or strange, even, so long as they are creative uses rather than ordinary uses. You may type in as many ideas as you can, both creative quality and quantity matter. Thus, it’s important to generate as many creative, unusual, and clever ideas as possible! You have 2 min to respond to each object.

AI: In the next message we will tell you an object. The goal is to come up with creative ideas, which are ideas that strike people as clever, unusual, interesting, uncommon, humorous, innovative, or different. Your ideas don’t have to be practical or realistic; they can be silly or strange, even, so long as they are creative uses rather than ordinary uses. You may type in as many ideas as you can, both creative quality and quantity matter. Thus, it’s important to generate as many creative, unusual, and clever ideas as possible. You have 2 min to respond to each object.

## Appendix 2

See Tables 5 and 6.

**Table 5:** Descriptive statistics of each task’s mean and maximum subjective rating separated by approach and group.

		Mean subjective rating								Maximum subjective rating							
		Original				Modified				Original				Modified			
		*M*	*SD*	*Min*	*Max*	*M*	*SD*	*Min*	*Max*	*M*	*SD*	*Min*	*Max*	*M*	*SD*	*Min*	*Max*
BardAI	Rope					1.95	0.32	1.35	2.37					2.80	0.49	2.00	3.50
	Box					2.79	0.27	2.33	3.15					3.70	0.32	3.00	4.00
	Pencil					2.93	0.21	2.62	3.35					4.07	0.37	3.50	5.00
	Candle					2.03	0.23	1.40	2.40					3.10	0.43	2.50	4.00
CopyAI	Rope	2.24	0.39	1.50	2.83	2.04	0.21	1.65	2.45	2.73	0.52	2.00	3.50	2.90	0.34	2.50	3.50
	Box	2.66	0.45	1.83	3.13	2.66	0.22	2.18	3.10	3.32	0.40	3.00	4.00	3.63	0.30	3.00	4.00
	Pencil	3.04	0.33	2.50	3.63	3.15	0.24	2.73	3.55	3.59	0.58	2.50	4.50	4.37	0.30	4.00	5.00
	Candle	1.75	0.42	1.00	2.33	2.16	0.36	1.67	3.00	2.45	0.72	1.00	3.50	3.37	0.48	2.50	4.00
ChatGPT3.5	Rope	1.91	0.73	0.00	2.50	2.22	0.13	1.97	2.43	2.50	0.95	0.00	3.50	3.07	0.26	2.50	3.50
	Box	2.92	0.45	2.00	3.50	2.88	0.24	2.30	3.20	3.50	0.32	3.00	4.00	4.00	0.33	3.50	4.50
	Pencil	3.20	0.20	2.88	3.50	3.24	0.18	3.00	3.61	3.73	0.41	3.00	4.50	4.67	0.24	4.50	5.00
	Candle	1.99	0.76	0.00	2.80	2.14	0.33	1.40	2.60	2.68	0.98	0.00	3.50	3.37	0.55	2.50	4.50
ChatGPT4	Rope	2.12	0.53	1.00	2.63	2.28	0.19	2.00	2.70	2.77	0.34	2.50	3.50	3.37	0.30	3.00	4.00
	Box	2.30	0.87	1.00	3.33	2.79	0.42	1.67	3.23	3.18	0.25	3.00	3.50	3.93	0.42	3.50	4.50
	Pencil	3.17	0.41	2.50	3.67	3.37	0.22	3.11	3.90	3.82	0.75	2.50	5.00	4.63	0.23	4.50	5.00
	Candle	2.58	0.63	1.67	3.50	2.26	0.33	1.55	2.75	3.55	0.42	3.00	4.50	3.93	0.32	3.50	4.50
AI total	Rope	2.09	0.57	0.00	2.83	2.12	0.25	1.35	2.70	2.67	0.65	0.00	3.50	3.03	0.41	2.00	4.00
	Box	2.63	0.65	1.00	3.50	2.78	0.30	1.67	3.23	3.33	0.35	3.00	4.00	3.82	0.37	3.00	4.50
	Pencil	3.14	0.32	2.50	3.67	3.17	0.26	2.62	3.90	3.71	0.59	2.50	5.00	4.43	0.37	3.50	5.00
	Candle	2.11	0.70	0.00	3.50	2.15	0.32	1.40	3.00	2.89	0.86	0.00	4.50	3.44	0.54	2.50	4.50
Human	Rope	1.70	0.70	0.50	3.83	1.95	0.44	1.25	3.25	2.29	0.86	1.00	4.50	2.96	0.81	1.50	5.00
	Box	2.16	0.78	0.33	3.50	2.41	0.56	1.00	3.90	2.61	0.78	1.00	4.00	3.40	0.80	1.00	4.50
	Pencil	2.33	0.90	0.00	3.83	2.58	0.75	1.00	4.00	3.00	1.02	0.00	4.50	3.63	0.83	1.00	5.00
	Candle	1.59	0.86	0.00	3.50	1.94	0.58	1.00	3.20	2.05	1.04	0.00	4.50	2.95	0.96	1.00	5.00

**Table 6:** Descriptive statistics of each task’s mean and maximum semantic distance separated by approach and group.

		Mean semantic distance								Maximum semantic distance							
		Original				Modified				Original				modified			
		*M*	*SD*	*Min*	*Max*	*M*	*SD*	*Min*	*Max*	*M*	*SD*	*Min*	*Max*	*M*	*SD*	*Min*	*Max*
BardAI	Rope					0.95	0.04	0.86	1.01					1.04	0.03	0.96	1.12
	Box					0.97	0.02	0.94	1.00					1.06	0.03	1.00	1.10
	Pencil					0.97	0.02	0.92	1.01					1.03	0.01	1.02	1.05
	Candle					0.96	0.04	0.86	1.00					1.07	0.03	1.02	1.11
CopyAI	Rope	0.91	0.09	0.73	0.99	0.94	0.03	0.90	0.99	0.98	0.08	0.73	1.02	1.03	0.02	1.00	1.07
	Box	1.00	0.03	0.96	1.04	0.97	0.02	0.93	1.00	1.04	0.02	1.02	1.09	1.05	0.02	1.03	1.09
	Pencil	0.92	0.05	0.82	0.99	0.95	0.03	0.89	0.99	0.99	0.04	0.88	1.04	1.07	0.04	1.01	1.12
	Candle	1.00	0.05	0.86	1.05	0.97	0.04	0.86	1.01	1.04	0.02	1.00	1.07	1.06	0.02	1.03	1.10
ChatGPT3.5	Rope	0.95	0.04	0.86	1.00	0.95	0.03	0.90	0.99	1.01	0.03	0.91	1.03	1.03	0.02	1.01	1.08
	Box	0.98	0.05	0.87	1.03	0.96	0.03	0.88	1.00	1.02	0.06	0.87	1.09	1.06	0.03	1.00	1.11
	Pencil	0.97	0.05	0.82	1.01	0.92	0.04	0.84	0.98	1.00	0.06	0.82	1.04	1.10	0.02	1.06	1.12
	Candle	0.97	0.04	0.91	1.03	0.95	0.03	0.90	0.99	1.01	0.06	0.91	1.09	1.05	0.02	1.03	1.10
ChatGPT4	Rope	0.97	0.07	0.77	1.03	0.97	0.03	0.89	0.99	1.01	0.02	0.98	1.04	1.05	0.05	0.99	1.20
	Box	0.99	0.04	0.90	1.03	0.96	0.03	0.89	0.99	1.03	0.03	0.96	1.05	1.06	0.02	1.02	1.10
	Pencil	0.97	0.03	0.92	1.01	0.97	0.01	0.95	0.98	1.01	0.03	0.96	1.04	1.05	0.02	1.02	1.09
	Candle	0.99	0.02	0.95	1.02	0.95	0.03	0.89	1.02	1.02	0.02	0.97	1.04	1.06	0.02	1.04	1.11
AI total	Rope	0.94	0.07	0.73	1.03	0.95	0.03	0.86	1.01	1.00	0.05	0.73	1.04	1.04	0.03	0.96	1.20
	Box	0.99	0.04	0.87	1.04	0.96	0.02	0.88	1.00	1.03	0.04	0.87	1.09	1.06	0.03	1.00	1.11
	Pencil	0.95	0.05	0.82	1.01	0.95	0.03	0.84	1.01	1.00	0.05	0.82	1.04	1.06	0.04	1.01	1.12
	Candle	0.99	0.04	0.86	1.05	0.96	0.04	0.86	1.02	1.02	0.04	0.91	1.09	1.06	0.02	1.02	1.11
Human	Rope	0.91	0.10	0.69	1.08	0.93	0.06	0.73	1.02	0.97	0.08	0.74	1.11	1.03	0.04	0.87	1.10
	Box	0.93	0.07	0.80	1.05	0.95	0.05	0.83	1.02	0.99	0.08	0.82	1.08	1.04	0.05	0.87	1.11
	Pencil	0.91	0.10	0.66	1.05	0.93	0.07	0.70	1.03	0.98	0.09	0.66	1.06	1.01	0.05	0.76	1.08
	Candle	0.91	0.09	0.63	1.04	0.94	0.06	0.75	1.03	0.97	0.08	0.66	1.08	1.02	0.03	0.91	1.10

## Appendix 3

See Tables 7, 8, 9 and 10.

**Table 7:** Results of the simple mixed linear model with simple effects of the approach and fluency on the maximum semantic difference for the AI and the humans.

Fixed effects	AI				Human			
	β	t	df	*p*	β	t	df	*p*
Intercept	1.01	118.53	90.00	< 0.001	0.98	85.39	98.12	< 0.001
Approach	0.01	1.35	90.00	0.180	0.01	2.45	96.58	0.016
Fluency	0.00	2.72	90.00	0.008	0.00	2.29	97.13	0.024

Random effects	*Var*	*SD*			*Var*	*SD*		
Participant	0.00	0.01			0.00	0.03		

N_AI_ = 93, N_Observations AI_ = 372, N_Humans_ = 100, N_Observations Humans_ = 393.

**Table 8:** Results of the simple mixed linear model with simple effects of the group and fluency on the maximum semantic difference for the original and modified approaches.

Fixed effects	Original				Modified			
	β	t	df	*p*	β	t	df	*p*
Intercept	0.98	89.09	81.15	< 0.001	1.01	100.50	107.412	< 0.001
Group	0.02	3.51	79.71	< 0.001	0.00	1.07	107.00	0.287
Fluency	0.00	1.53	80.32	0.129	0.00	3.55	107.29	< 0.001

Random effects	*Var*	*SD*			*Var*	*SD*		
Participant								

N_Original_ = 83, N_Observations Original_ = 326, N_Modified_ = 110, N_Observations Modified_ = 439.

**Table 9:** Results of the simple mixed linear model with simple effects of the approach and fluency on the maximum subjective ratings for the AI and the humans.

Fixed effects	AI				Human			
	β	t	df	*p*	β	t	df	*p*
Intercept	2.78	19.74	369.00	< 0.001	2.56	15.21	97.69	< 0.001
Approach	− 0.12	− 1.33	369.00	0.185	0.26	3.10	96.30	0.003
Fluency	0.02	4.72	369.00	< 0.001	0.02	1.94	96.86	0.055

Random effects	*Var*	*SD*			*Var*	*SD*		
Participant	0.00	0.00			0.47	0.22		

N_AI_ = 93, N_Observations AI_ = 372, N_Humans_ = 100, N_Observations Humans_ = 394.

**Table 10:** Results of the simple mixed linear model with simple effects of the group and fluency on the maximum subjective ratings for the original and the modified approaches.

Fixed effects	Original				Modified			
	β	t	df	*p*	β	t	df	*p*
Intercept	2.55	19.40	80.65	< 0.001	2.81	14.33	106.60	< 0.001
Group	0.32	5.62	79.08	< 0.001	0.02	0.24	106.13	0.814
Fluency	0.02	2.36	79.85	0.021	0.02	3.38	106.46	0.001

Random effects	*Var*	*SD*			*Var*	*SD*		
Participant	0.08	0.29			0.11	0.32		

N_Original_ = 83, N_Observations Original_ = 327, N_Modified_ = 110, N_Observations Modified_ = 439.
