# Supplementary material for: Time-on-task and instructions help humans to keep up with AI: replication and extension of a comparison of creative performances
Source: Sci Rep. 2025 Jun 20;15:20173. doi: 10.1038/s41598-025-05745-z (PMC12181282; doi:10.1038/s41598-025-05745-z)
Supplement: Supplementary file 1 — Supplementary Material 1 [file 41598_2025_5745_MOESM1_ESM.docx]

**Supplement I – analysis of differences between AI**

**Analysis**

To elaborate on differences between the AIs and between the tasks, we calculated repeated measures ANCOVAs with the mean semantic difference, maximum semantic distance, mean subjective rating, and maximum subjective rating as the dependent variables. The instruction (original vs. modified) and the group (ChatGPT 3.5, ChatGPT 4, CopyAI, and humans) were entered as between subject factors. The task (rope, box, pencil, candle) was entered as a repeated measures factor. Please note that these analyses were conducted without BardAI as the values for original instructions were not available for this AI. Fluency was entered as a covariate for each analysis. Post-Hoc tests were conducted Holm-corrected.

**Differentiating performance between AI chatbots and objects: semantic distance**

The first repeated measures ANOVA was conducted with the mean semantic distance (Figure S1) as a dependent variable. A Mauchly’s test of sphericity indicated deviations from this prerequisite (W = 0.93, χ²(5) = 11.17, *p* = .048). We, thus, used Greenhouse-Geisser correction for all between subject effects. We found no effect of the task or any interaction of the task with fluency, group, instructions, or group and instruction on the mean semantic difference (Table S1).

**Table S1**

Results of the repeated measures ANCOVA on the mean and maximum semantic difference.

|  | *mean semDis* | | | *maximum semDis* | | |
| --- | --- | --- | --- | --- | --- | --- |
| Repeated measures effects | *F* | *df* | *p* | *F* | *df* | *p* |
| Task | 1.57 | 2.87 | .198 | 1.62 | 3 | .183 |
| Task x group | 1.45 | 8.61 | .169 | 1.27 | 9 | .250 |
| Task x instructions | 1.66 | 2.87 | .177 | 0.62 | 3 | .604 |
| Task x fluency | 1.06 | 2.87 | .366 | 1.71 | 3 | .165 |
| Task x group x instruction | 1.19 | 8.611 | .300 | 1.49 | 9 | .149 |
| Residuals |  | 467.86 |  |  | 489 |  |
| Between subjects effects | *F* | *df* | *p* | *F* | *df* | *p* |
| Group | 8.82 | 3 | < .001 | 2.24 | 3 | .085 |
| Instructions | 0.91 | 1 | .343 | < 0.01 | 1 | .991 |
| Fluency | 1.97 | 1 | .162 | 12.49 | 1 | < .001 |
| Group x instructions | 0.53 | 3 | .666 | 2.64 | 3 | .051 |
| Residuals |  | 163 |  |  | 163 |  |

Of the between subject effects, only the group had a significant effect on the mean semantic difference (*p* < .001). The instructions, fluency and the interaction of group and instructions did not become significant. Holm-corrected post-hoc tests showed that the human showed lower values than CopyAI (*p* = .001), ChatGPT 3.5 (*p* = .002) and CHatGPT 4 (*p* < .001). The average values of the AIs did not differ (all *p* > .05).

**Figure S1**

Boxplots for the mean semantic distance for each task for the AI types and human groups separate for the modified and the original instructions.


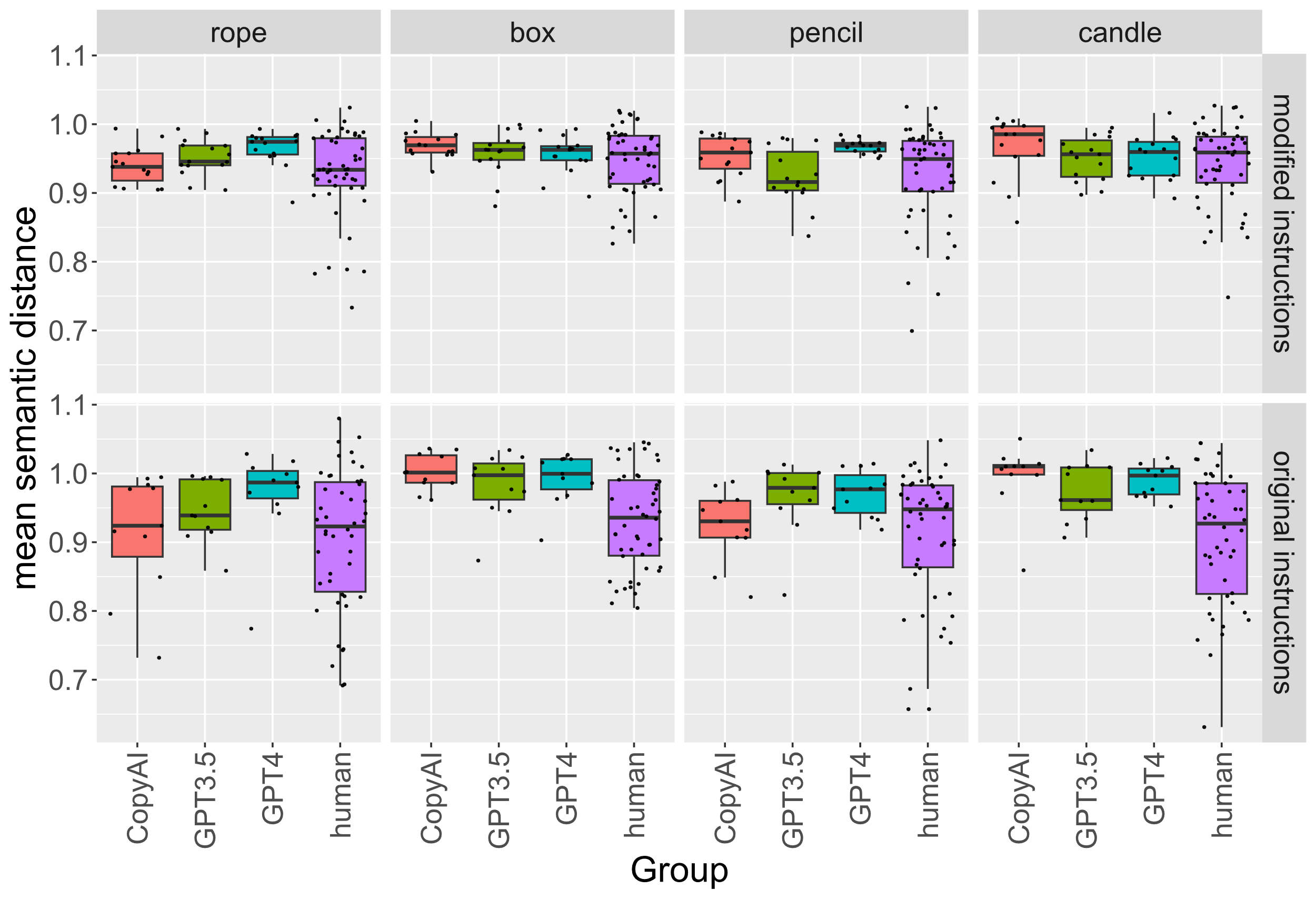


We further repeated this analysis using the maximum semantic distance as a dependent variable (Figure S2). A Mauchly’s test indicated that the assumption of sphericity was not violated (W = 0.94, χ²(5) = 8.89, *p* = .078). We found a main effect of fluency and a marginally significant interaction (Table S1). We decided to test for simple effects even though the interaction was only marginally significant (fluency was included for all conditional effects tests). Of the simple effects of the instruction for each of the groups (Table S2), we found a significant effect only for the human group.

**Table S2**

Results of the simple effect of the instructions on the maximum semantic difference for each of the group

| group | between subject effect | *F* | *df* | *p* |
| --- | --- | --- | --- | --- |
| CopyAI | approach | < 0.01 | 1 | .999 |
| ChatGPT3.5 | approach | 0.30 | 1 | .588 |
| ChatGPT4 | approach | 0.03 | 1 | .856 |
| Human | approach | 5.56 | 1 | .021 |

The humans showed significantly higher average values in the group receiving modified approach compared to the group receiving the original approach. The other simple effects showed that there was a significant effect of group for the original approach (*F*(3) = 4.07, *p* = .010) but not for the modified approach (*F*(3) = 1.89, *p* = .137). Holm-corrected post-hoc tests showed that the only significant difference was between ChatGPT4 and the human group (*p* = .034). On average, values were significantly higher for ChatGPT4 compared with humans participants. These findings, however, are dependent on the non-significant interactions which is close to being significant. If following the statistical conventions strictly, the results of this analysis would be different: No significant group differences or differences between the approach could be reported.

**Figure S2**

Boxplots for the maximum semantic distance for each task for the AI types and human groups separate for the modified and the original approach.


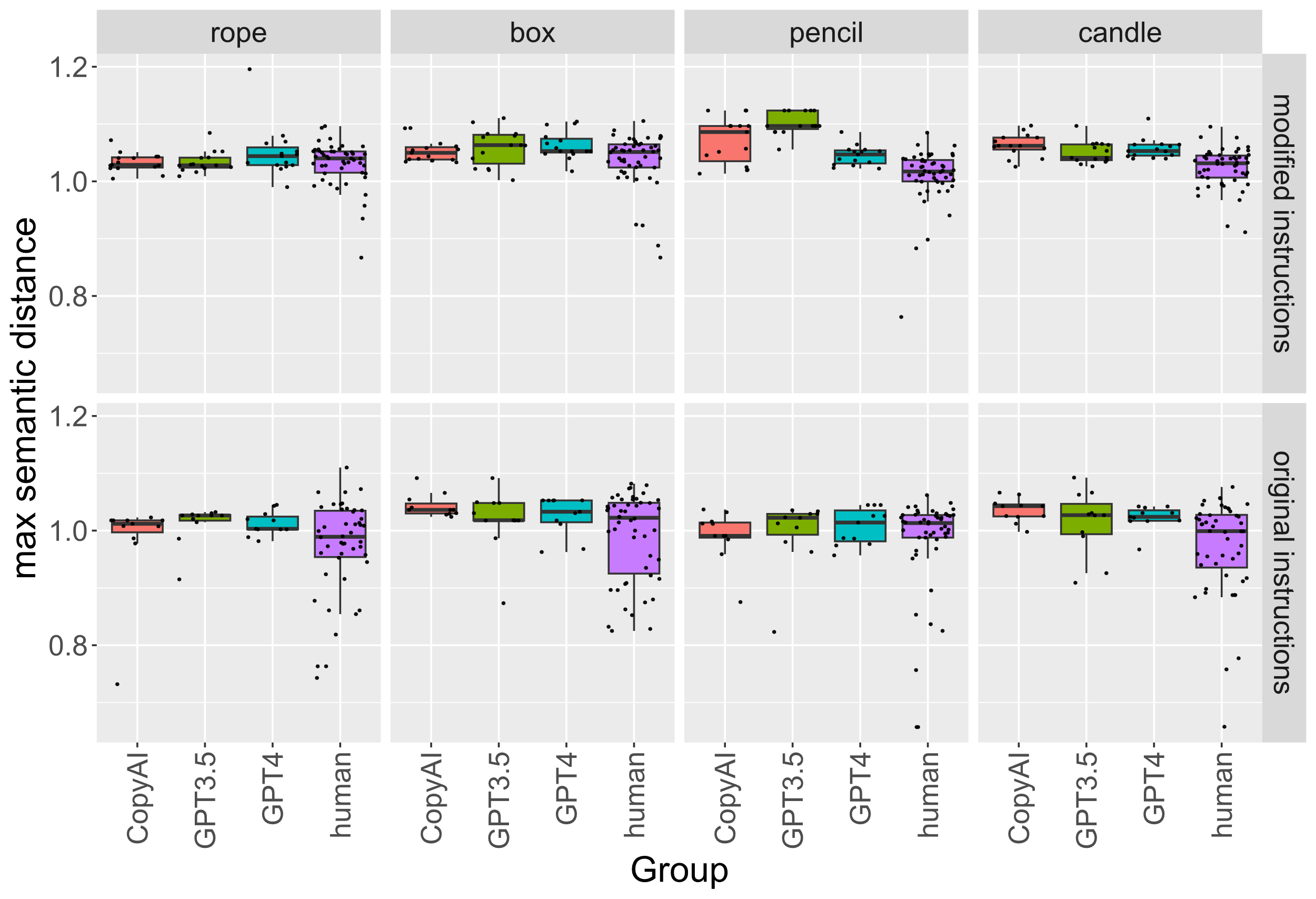


**Differentiating performance between AI chatbots and objects: subjective ratings**

We also calculated a repeated measures ANCOVA with the mean subjective rating as the dependent variable (Figure S3). We found no derivation from the assumption of sphericity in the Mauchly’s test (*W* = 0.97, χ²(5) = 4.75, *p* = .447) (Table S3).

**Table S3**

Results of the repeated measures ANCOVA on the mean and maximum subjective ratings.

|  | *mean subjective* | | | *maximum subjective* | | |
| --- | --- | --- | --- | --- | --- | --- |
| Repeated measures effects | *F* | *df* | *p* | *F* | *df* | *p* |
| Task | 7.90 | 3 | < .001 | 6.05 | 2.65 | < .001 |
| Task x group | 2.72 | 9 | 0.004 | 2.54 | 7.96 | 0.011 |
| Task x approach | 0.25 | 3 | 0.860 | 0.97 | 2.65 | 0.397 |
| Task x fluency | 0.26 | 3 | 0.857 | 2.45 | 2.65 | 0.071 |
| Task x group x approach | 1.72 | 9 | 0.083 | 1.06 | 7.96 | 0.394 |
| Residuals |  | 492 |  |  | 434.95 |  |
| Between subjects effects | *F* | *df* | *p* | *F* | *df* | *p* |
| Group | 12.68 | 3 | < .001 | 4.97 | 3 | 0.002 |
| Approach | 2.70 | 1 | 0.102 | 0.29 | 1 | 0.592 |
| Fluency | 0.68 | 1 | 0.410 | 9.12 | 1 | 0.003 |
| Group x approach | 0.13 | 3 | 0.943 | 2.23 | 3 | 0.087 |
| Residuals |  | 164 |  |  | 164 |  |

We found significant main effects for the task and the group that were qualified by a significant interaction of both factors. We tested for conditional effects including fluency as a covariate. Of all groups, we found significant simple main effects only for ChatGPT 3.5 and the human group (Table S4).

**Table S4**

Results of the simple effect of the task on the mean subjective rating for each of the group

| group | within subject effect | *F* | *df* | *p* |
| --- | --- | --- | --- | --- |
| CopyAI | task | 18.88 | 3 | < .001 |
| ChatGPT3.5 | task | 22.15 | 1.79 | < .001 |
| ChatGPT4 | task | 6.97 | 2.12 | .002 |
| Human | task | 8.62 | 2.79 | < .001 |

Candle and rope did not differ for all groups (all p > .05). In addition, box and pencil (*p* = .129) were not scored significantly different for humans and box and candle were for ChatGPT4 (*p* = .334). All other differences were significant (all other *p* < .001).

We also tested the simple main effects of the group for each of the tasks. We found significant simple main effects of the group for each task (Table S5). For the task rope, humans scored only marginally significantly lower than ChatGPT4 (*p* = .066). For box, humans scored sig. lower than ChatGPT3.5 (*p* < .001). For candle, humans scored sig. lower than ChatGPT 4 (*p* = .002). For pencil, humans scored significantly lower than all AI (all *p* < .001).

**Table S5**

Results of the simple effect of the group on the mean subjective rating for each of the tasks

| group | between subject effect | *F* | *df* | *p* |
| --- | --- | --- | --- | --- |
| rope | group | 3.16 | 3 | .026 |
| box | group | 5.98 | 3 | < .001 |
| candle | group | 4.52 | 3 | .004 |
| pencil | group | 5.62 | 3 | < .001 |

**Figure S3**

Boxplots for mean subjective rating semantic distance for each task for the AI types and human groups separate for the modified and the original approach.


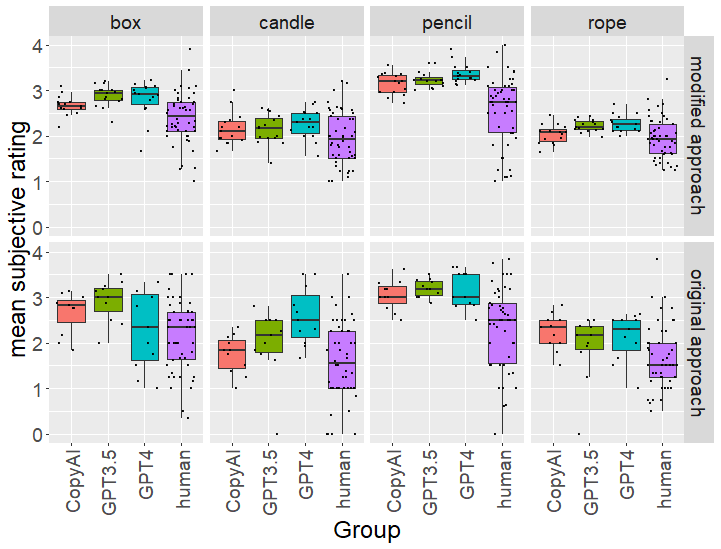


As a last analysis of study 1, we calculated a repeated measures ANCOVA with the maximum subjective rating as the dependent variable (Figure S4). As before, the group and the approach were added as factors. The Mauchly’s test (*W* = 0.83, χ²(5) = 31.08, *p* < .001) indicated no sphericity in the data. We thus used the Greenhouse-Geisser correction. We found a significant main effect for the task, a significant interaction of task and group, a significant main effect for the AI type, and significant main effect for fluency. To qualify the significant interaction of task and group, we tested the conditional effects (fluency was included as a covariate). There are significant differences in the maximum subjective rating between all tasks (*p* < .05) except rope and candle (*p* = .298) for humans. Further, we found differences between all tasks (*p* < .001) except between box and candle (*p* = .167) for ChatGPT4, between candle and rope for ChatGPT3.5 (*p* = .054), and CopyAI (*p* = .153) (Table S6).

We further found significant conditional effect of the groups for box, candle, and pencil (Table S7). Again, fluency was included as a covariate. For the task box, humans scored significantly lower compared to ChatGPT3.5 (*p* = .004). For candle, humans scored significantly lower than ChatGPT 4 (*p* = .001). For pencil, humans were significantly worse than ChatGPT3.5 (*p* = .005).

**Table S6**

Results of the simple effect of the task on the maximum subjective rating for each of the group

| group | within subject effect | *F* | *df* | *p* |
| --- | --- | --- | --- | --- |
| CopyAI | task | 12.09 | 3 | < .001 |
| ChatGPT3.5 | task | 11.02 | 2.21 | < .001 |
| ChatGPT4 | task | 9.73 | 3 | < .001 |
| Human | task | 7.49 | 2.58 | < .001 |

**Table S7**

Results of the simple effect of the group on the maximum subjective rating for each of the tasks

| task | between subject effect | *F* | *df* | *p* |
| --- | --- | --- | --- | --- |
| rope | group | 0.39 | 3 | .759 |
| box | group | 4.31 | 3 | .006 |
| candle | group | 4.76 | 3 | .003 |
| pencile | group | 5.27 | 3 | .002 |

**Figure S4**

Boxplots for maximum subjective rating semantic distance for each task for the AI types and human groups separate for the modified and the original approach.


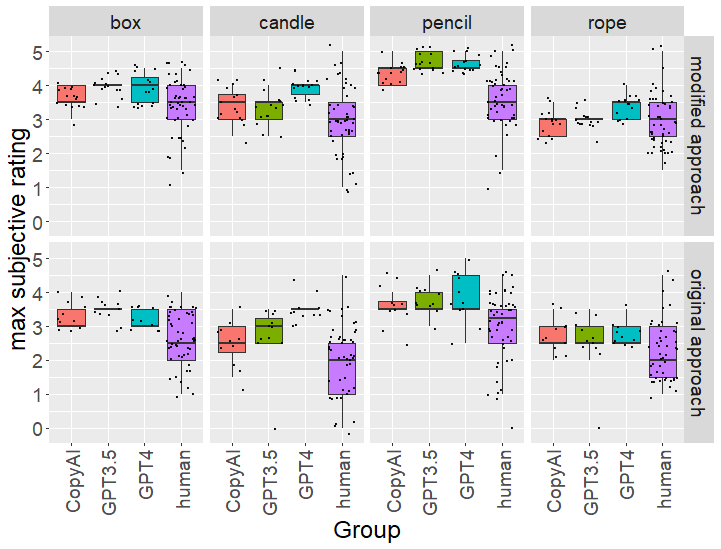


**Supplement II – Analysis of variance differences**

Following an anonymous reviewer’s suggestion after inspecting the figures in the main document, we exploratorily tested for differences in variance for all four measures (mean and maximum semantic distance and subjective ratings). For this purpose, we calculated the absolute mean difference for each case and measure. For each of the four measures, we ran a linear mixed model analysis using JASP Version 0.18.3.0^49^. The approach (original = 0 vs. modified = 1), group (human = 0 vs. AI = 1), and their interaction were added as fixed effects. The participant ID was added as a random effect grouping variable.

**Table S9**

Results of the mixed linear model on the absolute mean difference of the mean and maximum semantic distance.

|  | *mean semantic distance* | | | | *maximum semantic distance* | | | | |
| --- | --- | --- | --- | --- | --- | --- | --- | --- | --- |
| Fixed Effects | β | *t* | *df* | *p* | | β | *t* | *df* | *p* |
| Intercept | 0.05 | 26.65 | 187.56 | < .001 | | 0.04 | 23.27 | 190.25 | < .001 |
| Approach | -0.01 | -6.34 | 187.56 | < .001 | | -0.01 | -5.86 | 190.25 | < .001 |
| Group | -0.01 | -8.13 | 187.56 | < .001 | | -0.01 | -6.33 | 190.25 | < .001 |
| Approach x Group | 0.00 | 1.79 | 187.56 | 0.075 | | 0.01 | 3.75 | 190.25 | < .001 |
| Random Effects | *Var* | *SD* |  |  | | *Var* | *SD* |  |  |
| Participant | 0.00 | 0.01 |  |  | | 0.00 | 0.01 |  |  |

We found main effects for the Approach and Group for both measures, and a significant interaction for the maximum semantic distance. The absolute mean difference of the mean semantic distance was significantly higher for the humans compared to the AI and for the original compared to the modified approach. Due to the significant interaction for the absolute mean difference of the maximum semantic distance, we calculated simple effects. We found significant simple effects of the approach for the human group (β = 0.02, *t*(98.68) = 5.30, *p* < .001) and the AI (β = 0.02, *t*(91) = 8.97, *p* < .001). We also found effects of the group for the modified approach (β = 0.02, *t*(108.20) = 6.17, *p* < .001) and for the original (β = 0.02, *t*(108.20) = 3.53, *p* < .001). Together, these findings indicate that while all differences are significant, the effects of the approach on the absolute mean difference of the maximum semantic distance seems to be bigger for the humans compared to the AI. Similarly, the difference in absolute mean difference between human and AI seems to be bigger for the original approach compared to the modified approach (*Table S10*).

**Table S10**

Estimated marginal means of the absolute mean difference of the mean and maximum semantic distance by Approach and Group.

| Approach | Group | *mean semantic distance* | *maximum semantic distance* |
| --- | --- | --- | --- |
| modified | AI | 0.024 | 0.024 |
| original | AI | 0.040 | 0.030 |
| modified | human | 0.046 | 0.032 |
| original | human | 0.075 | 0.062 |

We found significant main effects for approach and group but no interaction for the mean subjective ratings. For the maximum subjective rating, we found a significant main effect for the group but not for the approach. We further did not find a significant interaction (*Table S11*). Marginal means show higher absolute mean differences for the human participants compared to the AI for both measures. IN addition, values are higher in the original approach for the mean subjective rating (*Table S12*).

**Table S11**

Results of the mixed linear model on the absolute mean difference of the mean and maximum subjective ratings.

|  | *mean subjective ratings* | | | | *maximum subjective ratings* | | | | |
| --- | --- | --- | --- | --- | --- | --- | --- | --- | --- |
| Fixed Effects | β | *t* | *df* | *p* | | β | *t* | *df* | *p* |
| Intercept | 0.57 | 35.56 | 187.22 | < .001 | | 0.66 | 30.91 | 189.53 | < .001 |
| Approach | -0.07 | -4.54 | 187.22 | < .001 | | -0.01 | -0.52 | 189.53 | 0.607 |
| Group | -0.06 | -3.79 | 187.22 | < .001 | | -0.10 | -4.67 | 189.53 | < .001 |
| Approach x Group | 0.02 | 0.92 | 187.22 | 0.359 | | 0.02 | 0.79 | 189.53 | 0.433 |
| Random Effects | *Var* | *SD* |  |  | | *Var* | *SD* |  |  |
| Participant | 0.02 | 0.12 |  |  | | 0.03 | 0.16 |  |  |

**Table S12**

Estimated marginal means of the absolute mean difference of the mean and maximum subjective ratings by Approach and Group.

| Approach | Group | *mean subjective ratings* | *maximum subjective ratings* |
| --- | --- | --- | --- |
| modified | AI | 0.453 | 0.56 |
| original | AI | 0.569 | 0.55 |
| modified | human | 0.545 | 0.73 |
| original | human | 0.721 | 0.78 |
